# Unmanned aerial vehicle (UAV) images of road vehicles dataset

**DOI:** 10.1016/j.dib.2024.110264

**Published:** 2024-03-02

**Authors:** Nama Ezzaalddin Mustafa, Fattah Alizadeh

**Affiliations:** a School of Science and Engineering, University of Kurdistan-Hewler, Erbil, Iraq; b Department of Computer Science, University of Toronto Metropolitan, Toronto, Canada

**Keywords:** UAV, Vehicle detection, Vehicle classification, Vehicle tracking, Data augmentation, Machine learning, Deep learning

## Abstract

The Intelligent Transportation System (ITS) seeks to improve traffic flow to guarantee transportation safety. One of the ITS's fundamental tenets is identifying and classifying vehicles into various classes. Although the issues related to small size, variety of forms, and similarity in visual appearance of the vehicles, as well as the influence of the weather on the video and image quality, make it challenging to categorize vehicles using unmanned aerial vehicles (UAV); they are becoming more popular in computer vision-related applications. Traffic accidents are now a serious public health concern that must be addressed in the Kurdistan Region of Iraq. An automatic vehicle detection and classification system can be considered one of the remedies to solve this issue. This paper presents a dataset of 2,160 images of vehicles on the roads in the Iraqi Kurdistan Region to address the issue of the absence of such a dataset. The images in the proposed collection were taken with a Mavic Air 2 drone in the Iraqi cities of Sulaymaniyah and Erbil. The images are categorized into five classes: bus, truck, taxi, personal car, and motorcycle. Data gathering considered diverse circumstances, multiple vehicle sizes, weather and lighting conditions, and massive camera movements. Pre-processing and data augmentation methods were applied to the images in our proposed dataset, including auto-orient, brightness, hue, and noise algorithm, which can be used to build an efficient deep learning (DL) model. After applying these augmentation techniques for the car, taxi, truck, motorcycle, and bus classes, the number of images was increased to 5,353, 1,500, 1,192, 282, and 176, respectively.

Specifications Table

**Table:** 

Subject	Computer Science, Artificial intelligence, Computer Vision, and Pattern Recognition
Specific subject area	Road Vehicle images from UAVs
Data format	Raw and annotated
Type of data	2D RGB images (.JPG)
Data collection	Mavic Air 2 drone was used to capture the images and videos in the dataset. The drone's height is kept between 100 and 150 meters above the ground during image capture and video recording. 1920 × 1080 pixels is the output resolution, and the total bitrate is 36180 kbps.
Data source location	Country: Iraq City: Erbil, and Sulaymaniyah
Data accessibility	Repository name: Zenodo Data identification number: (10.5281/zenodo.7401615) Direct URL to data: https://zenodo.org/record/7401615

## 1 Value of the Data

• The dataset is created explicitly for practitioners of machine learning and deep learning.
• The dataset is used mainly for the automatic segmentation and classification of vehicles using Deep Neural Network (DNN) and Machine Learning (ML) methods.
• The suggested UAV image collection can be used for vehicle counting purposes in images and videos.
  The proposed dataset can be used for real-time video detection.
• Additionally, multi-vehicle detection systems can benefit from it.

## 2 Background

Data plays a significant role in any machine learning or deep learning model, as no model can be built and trained without data. Machine learning algorithms need relatively large training datasets to learn how to perform non-trivial tasks of segmentation, classification, and tracking of the objects, and the low quality of the training data will hinder the performance ability of all machine learning approaches.

Creating high-quality image datasets under diverse environmental conditions, such as lighting and illumination, varied views, and day/night timings, is the first step in any deep learning model aiming at object processing in video or images. This research aims to introduce a new dataset for detecting and classifying vehicles in the Iraqi Kurdistan region. Due to the availability of low-cost image-capturing technology and user-friendly UAVs, the dataset has utilized aerial imagery as it becomes more accessible and well-liked in deep learning. Aerial images are widely employed for various purposes, particularly those involving intelligent transportation systems and vehicle-related tasks [1], [2], [3].

## 3 Data Description

**Fig. 1:**
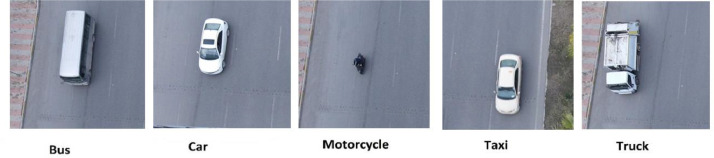
Types of vehicles in the proposed dataset.

The dataset discussed in this article comprises five distinct vehicle classes, as depicted in Fig. 1. These classes closely encompass the diverse range of vehicle types commonly found in the Kurdistan region.

Drone imagery of the vehicles passing on Kurdistan Region city streets and highways is the initial step in the dataset production procedure. Aside from that, we recorded videos of various vehicles, took frames from those recordings, and utilized them as images to simplify the procedure.

The images are distinct in many ways, with different types of automobiles in each class, varying lighting circumstances caught during the day and at night, shadows and haze in some images, and blurry images caused by the car's movement. Table 1 illustrates varying proportions of images captured under different conditions.

**Table 1:** Proportions of images in different categories.

Image Category	Proportion of Images (%)
Day	46%
Night	33%
Haze	19%
Shadow	5%
Blurry	27%

In addition, to increase the collection's diversity, the videos and images are captured in various surroundings, such as trees, buildings, et cetera. Also, various meteorological elements have been utilized, including rain, snow, and sunny days. Furthermore, the dataset poses difficulties for the peculiarities of aerial videos, such as varying vehicle sizes and shapes owing to altitude changes [4], [5], [6], [7].

**Fig. 2:**
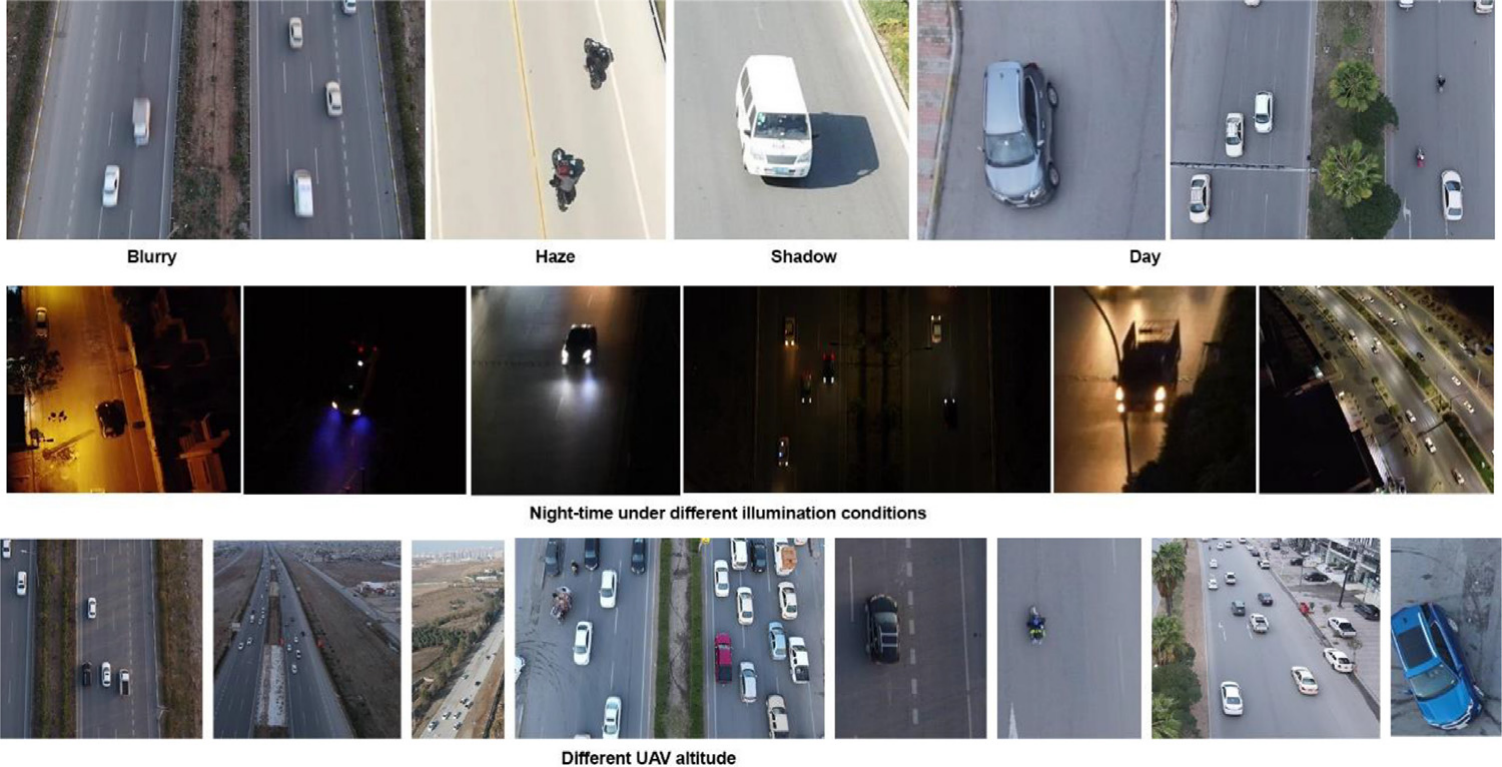
Vehicle images in various outside environments.

Fig. 2 shows a few example images that were shot under various circumstances.

The collection consists of 2160 images taken by drones flying above the cities of Erbil and Sulaymaniyah. In addition, for a reliable machine learning model, we partitioned the dataset into training, validation, and test sets to avoid bias and assure accurate model assessment. We employed 1509 images for the training set to adjust parameters, 430 images for the validation set to fine-tune hyperparameters, and the final 221 images for the test set to evaluate the optimized model.

**Fig. 3:**
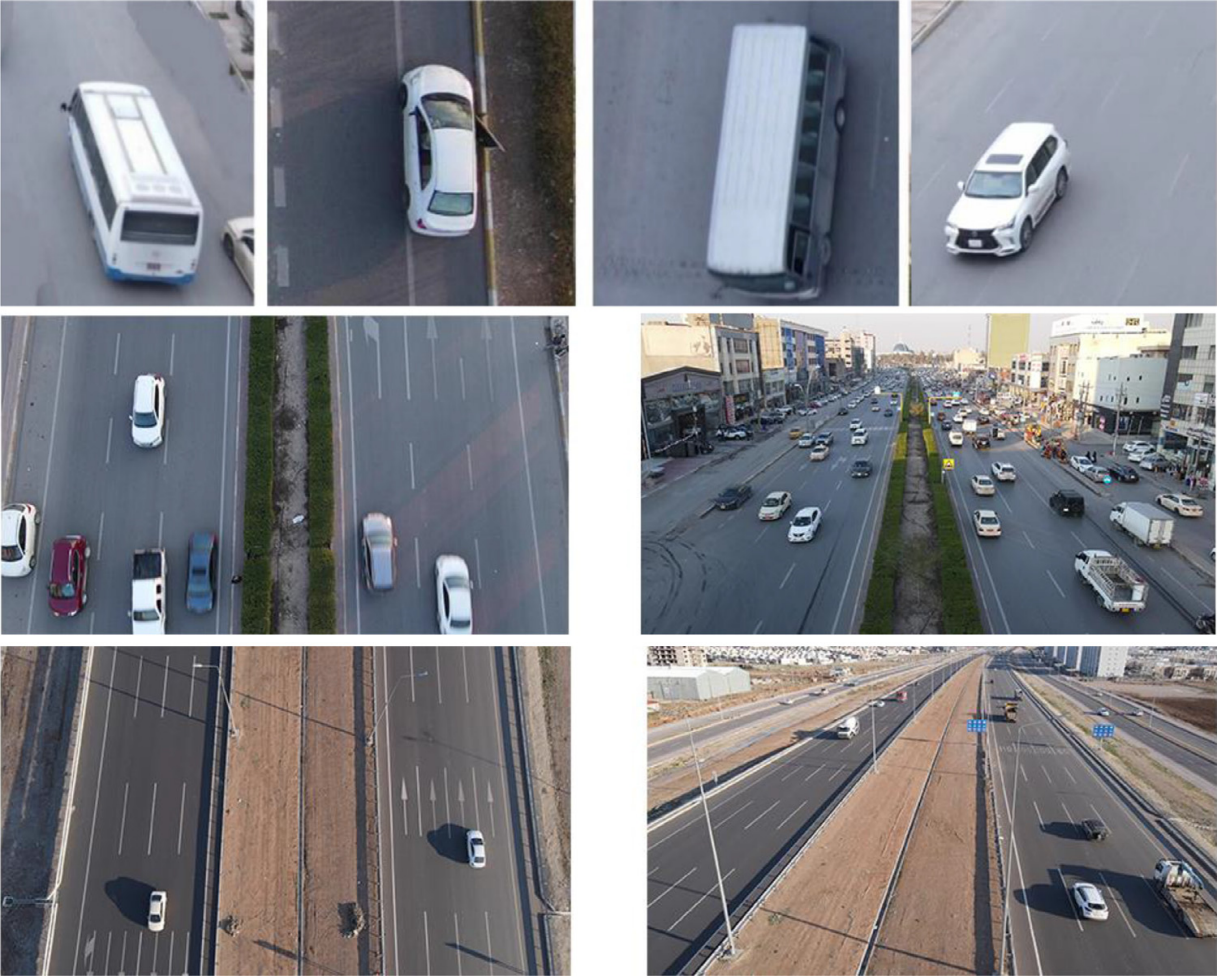
An example of vehicle images captured in several positions.

The dataset includes side views and top-down perspectives (bird's-eye view) images, allowing the system to recognize vehicles from all directions. This integration of different perspectives serves to enhance the training of robust object recognition models and provides a more comprehensive understanding of vehicles. By incorporating both side and top-down views, our dataset becomes versatile and applicable to a wide range of tasks. For instance, side views prove beneficial for vehicle classification [8], while top-down views offer value for tasks such as parking space detection [9]. An illustration of images from various view angles is shown in Fig. 3.

**Fig. 4:**
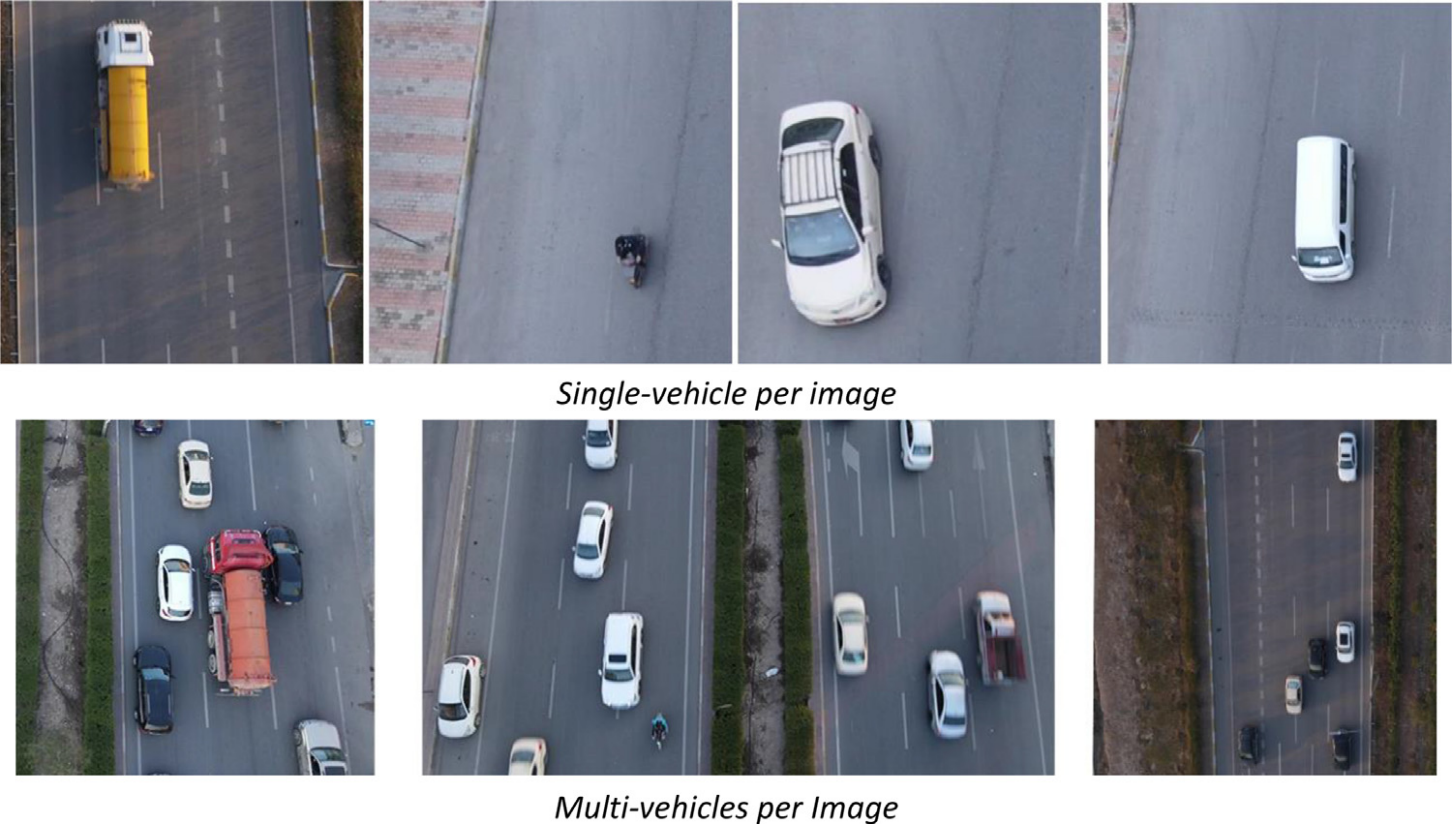
Single and multi-vehicle per image.

The dataset includes single-vehicle-per-image and multi-vehicle-per-image scenarios, as shown in Fig. 4.

Every image in the dataset has been carefully labeled with a particular vehicle class and accurately annotated with precise bounding boxes. The dataset covers five popular vehicle types in the Kurdistan area roadways (cars, taxis, trucks, buses, and motorcycles).

The dataset contains 2160 images of single and multiple vehicles per image; for each, there is a matching bounding box; the total number of annotations in the dataset is 8503, and they are categorized according to five different classes, as shown in Table 2.

**Table 2:** Number of annotated vehicles for each class.

Vehicle Class	Annotation Number
Car	5353
Taxi	1500
Truck	1,192
Motorcycle	282
Bus	176

**Fig. 5:**
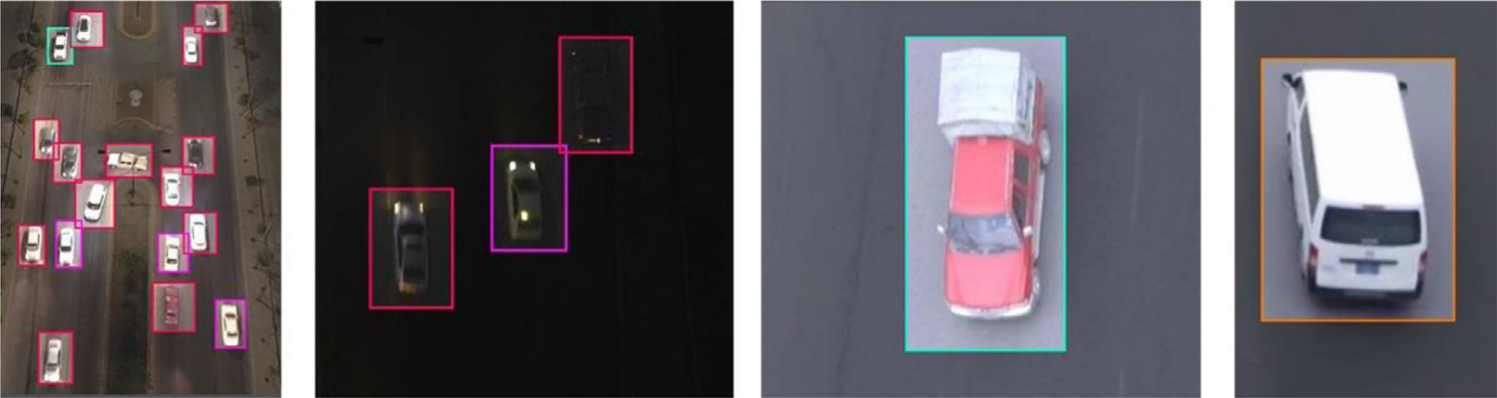
An example of annotation and labelling.

An example of annotation and labeling is shown in Fig. 5. Each vehicle class is represented using a different color.

We employed Faster R-CNN [10] and YOLO [11] object detection techniques as our baseline testing methods because of their exceptional performance in generic object detection. The outcomes, as outlined in Table 3, demonstrate the effectiveness of both algorithms on our dataset.

**Table 3:** Baseline results on our dataset.

Metric	Faster RCNN	YOLO
Motorcycle Accuracy	88.71	93.01
Bus Accuracy	93.91	92.21
Taxi Accuracy	97.65	87.06
Car Accuracy	71.11	78.07
Truck Accuracy	92.08	88.06
Overall Accuracy	88.69	87.68
Inference Speed (FPS)	4.16	35

Our baseline results suggest that both methods performed well on the dataset. YOLO is highly efficient, especially in terms of speed, as it processes the entire image in a single forward pass. This makes it ideal for real-time scenarios. Despite its amazing speed, YOLO tends to be somewhat less accurate than Faster R-CNN. Faster R-CNN, with its two-stage design, excels in accuracy but sacrifices speed compared to the swift performance of YOLO.

## 4 Experimental Design, Materials and Methods

### 4.1 Data Acquisition

#### 4.1.1 Specification of Data Preparation Device

The videos and images in the dataset were taken using a Mavic Air 2 drone. The drone's height was between 100 and 150 meters in the air when taking images and videos, although higher and lower altitudes were also considered. The resolution of the images is 1920 × 1080 pixels, and the overall bitrate is 36,180 kbps.

**Fig. 6:**
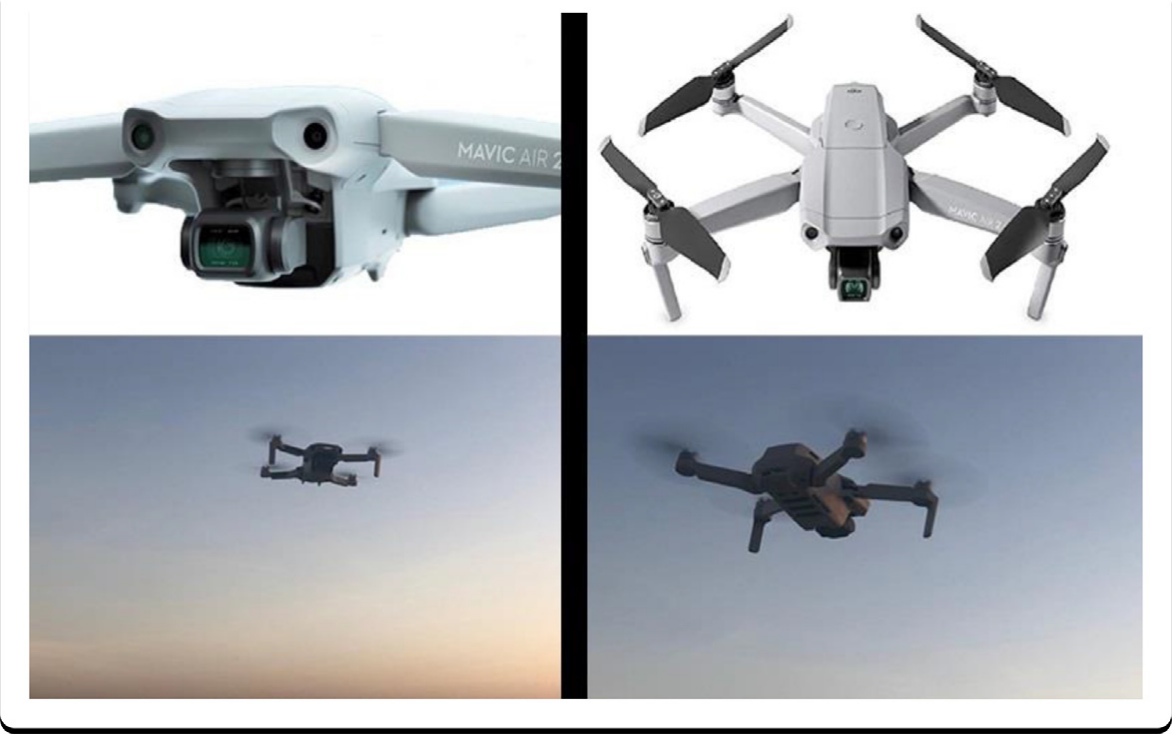
Mavic air 2 drone.

Fig. 6 shows the drone model (Mavic Air 2) utilized.

### 4.2 Data Cleaning

The cleaning was required as there were several possibilities for the images in the dataset to be duplicated or mislabeled due to the quality of the videos that the images extracted from.

Data were cleaned to correct or remove inaccurate, corrupted, improperly formatted, duplicate, or incomplete images. The dataset included several UAV images that either did not include any vehicles or only a portion of vehicles. Additionally, some images were unclear, making it challenging to identify the type of vehicle, owing to the UAV's high altitude or the drone's exposure to wind. The dataset also included some duplicate images which needed to be removed.

The dataset initially contained 3000 images; however, the data cleaning process led to the loss of 840 images (2160 remaining).

**Fig. 7:**
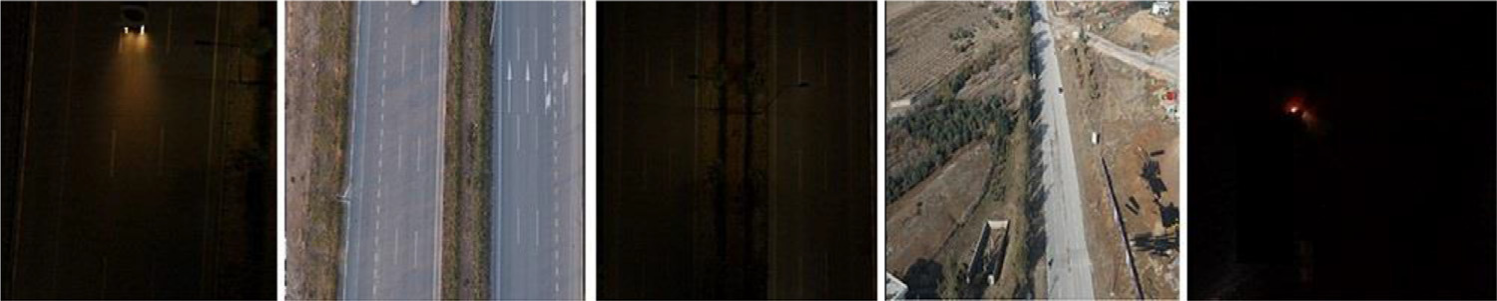
Some examples of removed images from the dataset.

Fig. 7 displays a few instances of the images that were deleted.

### 4.3 Data Pre-Processing

Data pre-processing is an essential phase of any training-based approach, and it would help the system to be trained faster using more relevant data. The data pre-processing steps will be explained in the sequel:

The dataset is divided into training, testing, and validation. These sets should be pre-processed to guarantee that training and prediction are performed on the same image features.

**Auto-orient:** Practically everyone puts their images into memory sideways without realizing it, and computers are not particularly good at detecting objects in sideways images. When the model fails to function correctly due to a sideways image loading, the programmer usually cannot identify the problem because most computer software only displays the image in its properly rotated form rather than how it is stored on disk. As a result, trying to see the image to find out why the model is not functioning well would fail since the picture will be shown correctly to the programmer, making it impossible to figure out the problem. The idea for solving this issue is to check for Exif Orientation information every time an image is imported into the Python scripts and rotate them, if necessary, by using Auto-orient, which disables the EXIF data from the images, allowing the system designer to view the images as they are saved on the disk such that they may get the right image and video orientation [12].

### 4.4 Data Augmentations

Data augmentation aims to get additional training data out of the existing dataset. The size of a dataset significantly influences the deep learning model's accuracy; more data typically leads to more robust and accurate models. When working with small datasets, models are more prone to overfitting. This difficulty can be solved via some techniques, among which data augmentation is more efficient and effective [13].

The following is the list of the transformations applied to the dataset to augment the data:

• **Brightness:** By adjusting the level of the intensity channel (in the HSI color model), it is possible to darken and brighten the images' brightness randomly. In this research, image brightness was improved by both brightening and darkening, making the applied learning algorithm more robust to changes in lighting and camera settings [14]. The intensity variation employed in this study to create the brighter and darker images is +25 for brightness and -25 for darkness.

Intensity_Brighter Image_ = Intensity_original Image_ + 25
Intensity_Darker Image_ = Intensity_original Image_ - 25

**Fig. 8:**
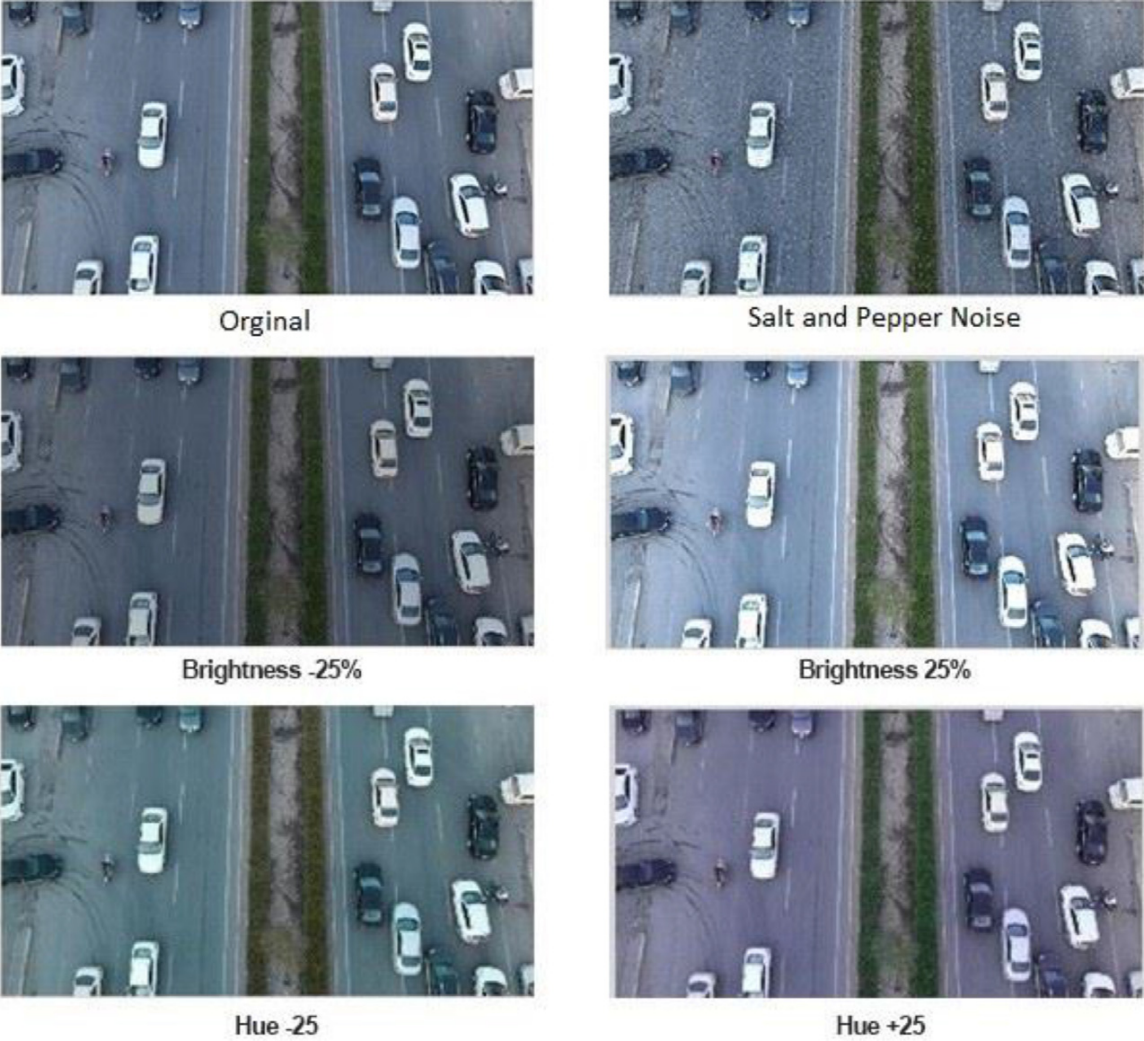
Data augmentation technique applied.
• **Noise:** The dataset also has been augmented by applying some noise; by adding 3% Salt and Pepper Noise.

• **Hue:** By randomly changing the colors of the input image, an underlying model may be able to classify a variety of color schemes for objects successfully. This approach is essential for preventing a model from memorizing a particular item's colors and allowing a model to consider object edges and forms rather than the images' simple color [14]. Images from the training set in our dataset will be augmented with hues in two distinct color ranges, as illustrated in Fig. 8.

Fig. 8 provides a sample of the data augmentation methods used on the dataset.

The specified data augmentation techniques were exclusively implemented on the training set, aiming to improve the model's generalization performance through increased diversity and the addition of more data. These methods have substantially expanded the training set, increasing the number of images from 1509 to 7545. When combined with the original validation set of 430 images and the testing set of 221 images, the total number of images in the dataset post-augmentation now comprises a total of 8196 images.

## Limitations

Challenges and Difficulties of the Data Collection Process.

It is important to note that the data collection was a challenging task due to the following issues while using the drone:

• **Security concern**: There are plenty of restrictions on using drones for image capturing in the Kurdistan region. Even though a permission letter was granted from the security authorities, there are still limitations to the use of drones.
• **Environmental issues**: The drone could not fly too high due to hazardous lasering from security locations, hotels, and other enterprises.
• **Human Hazards**: There were several cases of humans and kids stoning toward the drone, especially when the drone was flying far from its base location.
• **Lack of inter-diversity of vehicles**: Most vehicle types on the roads and highways in the Kurdistan Region are personal cars, with very few motorcyclists and bus classes. Class balance was, therefore, challenging to do.

## Ethics Statement

The authors affirm that when collecting data for the dataset proposed in this paper, we had informed consent from all drivers, that no number plates are visible, and that we had permission for the use of a drone.

## CRediT authorship contribution statement

**Nama Ezzaalddin Mustafa:** Software, Data curation, Methodology, Writing – original draft, Writing – review & editing. **Fattah Alizadeh:** Conceptualization, Visualization, Investigation, Supervision.

## Data Availability

UAV Images of Road Vehicles (Original data) (Zenodo).
